# Use of the Method of Triads in the Validation of Sodium and Potassium Intake in the Brazilian Longitudinal Study of Adult Health (ELSA-Brasil)

**DOI:** 10.1371/journal.pone.0169085

**Published:** 2016-12-28

**Authors:** Taísa Sabrina Silva Pereira, Nágela Valadão Cade, José Geraldo Mill, Rosely Sichieri, Maria del Carmen Bisi Molina

**Affiliations:** 1 Post Graduate Programme in Public Health, Federal University of Espírito Santo, Espírito Santo, Brazil; 2 Institute of Social Medicine, State University of Rio de Janeiro, Rio de Janeiro, Brazil; Leibniz-Institut fur Pflanzengenetik und Kulturpflanzenforschung Gatersleben, GERMANY

## Abstract

**Introduction:**

Biomarkers are a good choice to be used in the validation of food frequency questionnaire due to the independence of their random errors.

**Objective:**

To assess the validity of the potassium and sodium intake estimated using the Food Frequency Questionnaire ELSA-Brasil.

**Subjects/Methods:**

A subsample of participants in the ELSA-Brasil cohort was included in this study in 2009. Sodium and potassium intake were estimated using three methods: Semi-quantitative food frequency questionnaire, 12-hour nocturnal urinary excretion and three 24-hour food records. Correlation coefficients were calculated between the methods, and the validity coefficient was calculated using the method of triads. The 95% confidence intervals for the validity coefficient were estimated using *bootstrap* sampling. Exact and adjacent agreement and disagreement of the estimated sodium and potassium intake quintiles were compared among three methods.

**Results:**

The sample consisted of 246 participants, aged 53±8 years, 52% of women. Validity coefficient for sodium were considered weak (рfood frequency questionnaire actual intake = 0.37 and рbiomarker actual intake = 0.21) and moderate (рfood records actual intake 0.56). The validity coefficient were higher for potassium (рfood frequency questionnaire actual intake = 0.60; рbiomarker actual intake = 0.42; рfood records actual intake = 0.79). Conclusions: The Food Frequency Questionnaire ELSA-Brasil showed good validity in estimating potassium intake in epidemiological studies. For sodium validity was weak, likely due to the non-quantification of the added salt to prepared food.

## Introduction

The association of diet with non-communicable diseases (NCDs) has been observed in epidemiological studies [1], but estimates of a population's dietary intake is complex due to the diversity of instruments and errors pertaining to each method. The method most commonly used to assess population intake in longitudinal studies is the Food Frequency Questionnaire (FFQ), which, should reflect the characteristics of the population in question and should therefore be a validated instrument [2].

Validation of the FFQ with a reference method is essential for estimating errors caused by bias in the reporting of intake [2], although Food Records (FRs) or 24-hour Recall (R-24) [3], which are the most frequently used methods for this purpose, also present beyond random errors, recall bias and error in estimating portion sizes [4]. Biomarkers are a good choice to validate the FFQ due to the independence of their random errors in relation to dietary methods [5].

Biomarkers are classified into two types—recovery and concentration. Recovery ones accurately measure the intake and excretion of nutrients, whereas the concentration biomarkers are obtained by measuring serum micronutrient levels and urine electrolytes; these biomarkers cannot provide an absolute measure of food intake; rather, they provide correlations with intake levels [6]. One limitation of the use of biomarkers is reference time, as they reflect nutrient intake in the short term, i.e., intake at a point in time [4].

**Fig 1:**
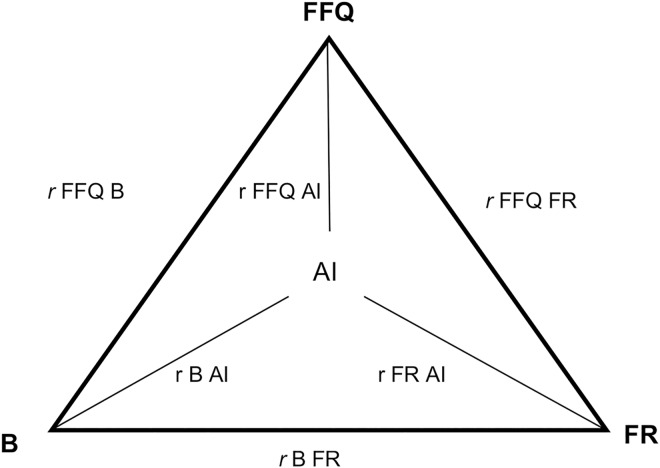
FFQ: food frequency questionnaire; FR: food record; B: biomarker (urinary excretion); AI: actual intake; *r* FFQ FR: bivariate correlation between food frequency questionnaire and reference method; *r* B FR: bivariate correlation between biomarker and reference method; *r* FFQ B: bivariate correlation between food frequency questionnaire and biomarker; r FFQ AI: validity coefficient of the food frequency questionnaire; r FR AI: validity coefficient of the reference method; r B AI: validity coefficient of the biomarker.

In light of this limitation, Kaaks [7] recommends using the triangulation technique or method of triads, as this method allows the comparison of food intake estimated by FFQ, FR and biomarker with actual intake (AI) through the use of the validity coefficient. The application of this technique requires that some assumptions be met, such as linearity of the correlation between the three methods and AI and the independence of random errors of the three [7]. Fig 1 shows the proposed analysis using the method of triads.

The validity coefficients (р) can be calculated using the formulas:

ρFFQAI=√(rFFQFRxrFFQB)∕rBFR

ρFRAI=√(rFFQFRxrBFR)∕rFFQB

ρBAI=√(rBFRxrFFQB)∕rFFQFR

Based on these formulas, correlation coefficients between the methods can be calculated:

rFFQFR=ρFFQAIxρFRAI

rFFQB=ρFFQAIxρBAI

rBFR=ρFRAIxρBAI

Thus, this study aims to assess the validity of sodium and potassium intake estimates using the FFQ ELSA-Brasil.

## Methods

### Study design and population

This quantitative, cross-sectional and analytical study was conducted with participants from the Longitudinal Study of Adult Health, ELSA-Brasil, aged 35–74 years, from six capital cities in three regions of Brazil: South (Federal University of Rio Grande do Sul), Southeast (University of São Paulo, Oswaldo Cruz Foundation, Federal University of Minas Gerais, Federal University of Espírito Santo) and Northeast (Federal University of Bahia), specifically from a subsample of 281 individuals who participated in the FFQ ELSA-Brasil validation study to thirteen nutrients [8]. ELSA-Brasil is a multicentre study; Approvals were granted by all institutional review boards: Sao Paulo University, Oswaldo Cruz Foundation, Federal University of Bahia, Federal University of Minas Gerais, Federal University of Espírito Santo, and Federal University of Rio Grande do Sul. All participants signed declarations of informed consent.

### Data collection

In terms of a sodium and potassium intake biomarker, ELSA-Brasil adopted the 12-hour nocturnal urine collection method as a tool to evaluate creatinine clearance and to estimate electrolyte intake (Na^+^, K^+^ and Ca^2+^) [9]. Participants were given verbal and written guidance from trained and certified personnel regarding the collection, storage and transportation of urine. Urinary volume was measured using a measuring cylinder with a capacity of 1000 mL and a precision of 10 mL. Sodium and potassium levels were measured using selective electrodes. Urine samples collected between 10 and 14 hours; urine output above 250 mL and for which there was no complete loss of urine were accepted. Urine samples were only considered valid when total creatinine excretion amounts, corrected by weight, were between 14.4 and 33.6 mg/kg in males and 10.8 and 25.2 mg/kg in females [10]. Participants who did not meet these criteria were excluded from the analysis.

The FFQ is a semi-quantitative questionnaire containing 114 food items that evaluates the participant's habitual intake over the last twelve months [11]. This FFQ is validated for the ELSA-Brasil population [8].

Regarding food record (FR) data, participants noted in detail their food intake over 24 hours at three time-points over the course of a year, with an interval of four months between the time-points. The first record was obtained in October 2009, and the second and third were obtained in March and August 2010, respectively. Participants were given verbal and written guidance on how to complete the FRs and received a full-size utensil set to help them quantify the portions/drinks ingested. Days of the week and weekend were represented according to the correct ratio (5:2), and all involved study centres performed the data collection simultaneously [8]. Participants who had energy intake values in FR and FFQ equal to or less than 500 kcal and greater than 6000 kcal [12] were excluded.

### Data analysis

The nutritional composition of the food items included in the FFQ and FR was estimated based on the American food composition table of the *Nutrition Data System for Research*—NDSR, of the University of Minnesota [13]. The Brazilian Food Composition Table (‘Tabela Brasileira de Composição de Alimentos’—TACO) of the State University of Campinas (Universidade Estadual de Campinas—UNICAMP) [14] was used for cassava flour. The nutritional compositions for regional preparations were calculated based on the individual components of each preparation according to recipes from technical literature from teaching and research institutions [8].

Based on the three food records intra-individual variability was estimated and individual energy and nutrient values de-attenuated using the method proposed by *Iowa State University* (ISU), using PC-SIDE software (Software for Intake Distribution Estimation for Windows OS) developed by the National Research Council of Iowa State University [15].

Nutrients were also adjusted by total energy intake, using the residual method proposed by Willett et al. [16]. However, the residual amount had a mean of zero, making it necessary to add a constant to the residual values. The constant represents nutrient intake for mean total energy intake of the studied population [6]. The α and β coefficients obtained from the regression were used to calculate the constant: C = α + (β * Mean energy of the group). The nutrient value adjusted for energy was thus determined.

To investigate the associations between FR, FFQ and urinary excretion data, raw and energy-adjusted partial correlation coefficients were calculated [16]. Logarithmic transformations were performed for data without normal distribution. Validity coefficients for the three methods–FR, FFQ and urinary excretion–were established using the method of triads [6].

The validity coefficients were classified as low (r < 0.2), moderate (r = 0.2–0.6) or high (r > 0.6) [17]. To calculate the 95% confidence interval of the validity coefficients, 1000 bootstrap samples of the same size as the study sample were generated using the R statistical package, version 3.0.2. The capacity of the FFQ to classify individuals into the same quintile of intake in relation to FRs and urinary excretion was assessed.

Data were analysed using the *Statistical Package for Social Sciences* (SPSS 17.0) statistical software (Chicago, USA, 2007), and the significance level adopted for all tests was α ≤ 5%.

## Results

From the initial sample of 281 participants, 29 were excluded for not meeting the urinary excretion criteria and six for not meeting the energy intake criterion. Thus, the final sample consisted of 246 participants with a mean age of 53 ± 8 years. Analysis stratifying by sex did not change materially the results.

Table 1 shows the sociodemographic characteristics and nutritional status according to the participant's gender. The proportions of each stratum by gender were similar.

**Table 1:** Sociodemographic characteristics and nutritional status of a subsample of participants in the Longitudinal Study of Adult Health, ELSA-Brasil, 2008–2010, according to gender.

	Gender	
	Male	Female
	(n = 118)	(n = 128)
	n (%)	n (%)
**Age Group (years)**		
35 to 44	26 (22.0)	17 (13.3)
45 to 54	37 (31.4)	51 (39.8)
55 to 64	44 (37.3)	50 (39.1)
65 to 74	11 (9.3)	10 (7.8)
**Income quintile**		
1^st^ quintile	17 (14.4)	23 (18.1)
2^nd^ quintile	37 (31.4)	25 (19.7)
3^rd^ quintile	20 (16.9)	31 (24.4)
4^th^ quintile	27 (22.9)	32 (25.2)
5^th^ quintile	17 (14.4)	16 (12.6)
**Nutritional status**		
Underweight/Normal	44 (37.3)	43 (33.6)
Overweight	49(41.5)	51 (39.8)
Obesity	25 (21.2)	34 (26.6)

Table 2 shows the mean sodium and potassium intake means estimated using the different methods. The three methods provided estimates of sodium intake above those recommended (2300 mg), while the opposite was true for potassium intake (4700 mg).

**Table 2:** Arithmetic means and standard deviations for sodium (mg) and potassium (mg) intake estimated from the Food Records, Food Frequency Questionnaire and Urinary Excretion of participants in the Longitudinal Study of Adult Health, ELSA-Brasil, 2008–2010.

Measurement method	Nutrient	
	Sodium (mg/day)	Potassium (mg/day)
	Mean±SD	Mean±SD
	(n = 246)	(n = 246)
***Urinary excretion***	4483±2198	2508±1047
***Food records***		
Raw	3344±982	3153±874
De-attenuated^¥^	3350±546	3205±564
De-attenuated and adjusted*	2044±424	1502±359
***Food frequency questionnaire***		
Raw	4657±1826	4919±1948
Adjusted*	4373±658	4680±1066

^¥^proposed by *Iowa State University*

* adjusted for energy

The Pearson correlations between each pair of methods are presented in Table 3 and were linear and positive, which justified using the method of triads. The correlations for sodium were low between the three methods but were significant between the adjusted FR x FFQ (r = 0.21, p < 0.01). For potassium, adjusted R x FFQ (r = 0.47, p < 0.01), FR x urinary excretion (r = 0.33, p < 0.01) and FFQ x urinary excretion (r = 0.25, p < 0.01) were all significant.

**Table 3:** Pearsons Correlations (r) between the methods intake estimated sodium and potassium for participants in the Longitudinal Study of Adult Health, ELSA-Brasil, 2008–2010.

Pearsons Correlation (r)	Nutrient	
	Sodium	Potassium
*FR x FFQ		
*r* Raw	0.43	0.25
*r* Adjusted	0.23	0.47
*FR x Urinary excretion		
*r* Raw	0.19	0.30
*r* Adjusted	0.12^¥^	0.33
**FFQ x Urinary excretion**		
*r* Raw	0.10^¥^	0.26
*r* Adjusted	0.08^¥^	0.25

FR–Food records; FFQ–Food frequency questionnaire; * for raw FR, the de-attenuated value was considered; *r–*correlation coefficient;

¥ Not significant

Table 4 shows the correlation coefficients, validity coefficients and the variation in validity coefficients. The validity coefficients for sodium were considered moderate (рFFQ AI [0.37]; рFR AI [0.56]) and weak (рB AI [0.21]); thus, the validity coefficients were higher for the dietary intake methods than for the biomarker. The validity coefficients for potassium were considered moderate (рFFQ AI [0.60]; рB AI [0.42]) and high (рFR AI [0.79]), and the dietary intake methods were superior to the biomarker.

**Table 4:** Correlation coefficients, validity coefficients, validity coefficients with Bootstrap 95% confidence interval and variations in validity coefficients for sodium and potassium, measured from the Food Frequency Questionnaire, Food Records and Urinary Excretion methods for participants in the Longitudinal Study of Adult Health, ELSA-Brasil, 2008–2010.

	Sodium	Potassium
**Correlation coefficient**		
*r*FFQ FR	0.23	0.47
*r*FFQ B	0.08	0.33
*r*B FR	0.12	0.25
**Validity coefficient**	Validity coefficient^†^ (95% CI)	Validity coefficient ^†^ (95% CI)
рFFQ AI	0.37 (0.08–1.00)	0.60 (0.41–0.74)
рFR AI	0.56 (0.16–1.00)	0.79 (0.61–1.00)
рB AI	0.21 (0.02–0.37)	0.42 (0.25–0.56)

*r* FFQ FR: correlation between food frequency questionnaire and reference method; *r* B FR: correlation between biomarker and reference method; *r* FFQ B: correlation between food frequency questionnaire and biomarker; ρFFQ AI: validity coefficient of food frequency questionnaire; ρFR AI: validity coefficient of the reference method; ρB AI: validity coefficient of the biomarker.

^†^ Values > 1.00 were considered to be 1.00.

Table 5 shows the exact and adjacent agreement and disagreement of the estimated sodium and potassium intake quintiles for the three methods, the exact agreement percentages ranged from 22.8 to 28% for sodium and from 22.8% to 29.7% for potassium.

**Table 5:** Agreement of estimated sodium and potassium intake between the Food Frequency Questionnaire, Food Records and Urinary Excretion methods for participants in the Longitudinal Study of Adult Health, ELSA-Brasil, 2008–2010.

	% Agreement		
	Exact	Adjacent	Disagreement
**Sodium**			
FFQ x FR	28.0	39.4	32.6
FFQ X B	22.8	32.5	44.7
FR x B	26.8	32.5	40.7
**Potassium**			
FFQ x FR	29.7	29.6	40.7
FFQ X B	25.2	33.8	41.0
FR x B	22.8	41.4	35.8

The nutrients were considered in raw form.

## Discussion

This study showed that the validity coefficients for potassium and sodium were moderate, with the exception of the sodium biomarker, whose validity coefficient was weak. No negative correlations were detected; the presence of negative correlations precludes the estimation of the validity coefficients. The dietary method validity coefficients performed better than the biomarker validity coefficient. Moreover, the 95% confidence intervals calculated using the bootstrap method showed large intervals for some coefficients, indicating low correlations between the studied methods [7].

Low correlation between the methods were also reported by Dia et al.[18] in a study with 123 participants, using urinary excretion of 24 hours, FFQ and food diary for seven days. FR and the FFQ have better correlations due to similar measurement errors.

Few studies have applied the method of triads to validate sodium and potassium intake, with the most frequent use being to validate nutrients such as tocopherols [19], carotenes [17, 20], fatty acids, protein [17,19], folic acid, vitamin B_12_ and fruit and vegetable intake [21]. Comparison between the validity coefficients of different studies should be interpreted with caution because the reference method used and the number of applications of each instrument can be different. In addition, other aspects must also be considered, such as sample size and number of food items on the FFQ [22].

In a study conducted in Costa Rica, Kabagambe et al. [23] used the method of triads to validate a FFQ for seven nutrients, including α-tocopherol, β-carotene and lycopene. They observed that the validity coefficients for some nutrients were higher for dietary methods and lower for biomarkers, corroborating the findings of the present study.

To estimate carotenoid and vitamin E intake, McNaughton et al. [24] attempted to validate a self-administered, semi-quantitative FFQ with 129 food items. They used a weighed food record over 2 non-consecutive days as the reference method and collected blood samples for the biomarker. The results revealed lower validity coefficients for the biomarkers.

In a validation study for an FFQ for fruit and vegetable intake, Andersen et al. [25] used serum carotenoids as a biomarker and used weighed food records over 14 days as the reference method. The highest validity coefficient was estimated using the reference method, with coefficients of р0.77 for vegetable intake and р0.79 for fruit and vegetable intake.

Mirmiram et al. [19] assessed different nutrients based on plasma and potassium contents using 24-hour urinary excretion and reported a validity coefficient for the biomarker of рB = 0.38, a value similar to that found in this study. Agreement values between the reference method and FFQ were stratified by gender, with figures of 50% and 39.8% being obtained for males and females, respectively.

To validate an FFQ estimate of fruit, juices and vegetable intake, Carlsen et al. [20] used seven 24-hour weighed food records. In terms of biomarkers, they used blood plasma to validate carotenoids and urinary excretion to validate flavonoids. Lower validity coefficients were observed for the biomarkers. The instrument was validated, as it was able to provide estimates similar to the reference method.

The combined use of biomarkers and FRs strengthens FFQ validation, but the method of triads has its limitations. Such limitations include the 95% confidence intervals are greater than one [23]; the method of triads is based on the assumption that the errors associated with each of the three methods included in the model are independent; and the method of triads assumes that the FFQ, FR and biomarkers have linear relationships with actual intake [17].

The traditional methods for assessing food intake performed better than the biomarkers, indicating that the biomarker should only be used as a complementary measure and not in place of dietary surveys. In this study, FFQ and urinary excretion were collected at the same time; however, FFQ expresses habitual intake over the last twelve months, and urinary excretion represents daily intake. The FRs were completed approximately one year after urine measurement, i.e., the three FRs were performed at four-month intervals, each indicating an intake time-point. Despite these different time periods, the validity coefficients were moderate. These results demonstrate that for some nutrients, dietary assessment methods are better.

Our findings are in line with results of intake of sodium and potassium obtained by the FFQ compared with biomarkers from five large studies that concluded that the use of potassium can well reflect the real intake when this nutrient is adjusted by the energy obtained by doubly-labeled water, while the values from sodium reflects less the real intake [26].

## Conclusion

The FFQ—ELSA-Brasil is a good instrument in estimating potassium intake in epidemiological studies but it is less reliable for sodium intake, whereas FR performed well for both potassium and sodium.

## Supporting Information

S1 Data Data is available in Data plos one2.sav (SAV)
